# Mediation of Autophagic Cell Death by Type 3 Ryanodine Receptor (RyR3) in Adult Hippocampal Neural Stem Cells

**DOI:** 10.3389/fncel.2016.00116

**Published:** 2016-05-06

**Authors:** Kyung Min Chung, Eun-Ji Jeong, Hyunhee Park, Hyun-Kyu An, Seong-Woon Yu

**Affiliations:** Department of Brain and Cognitive Sciences, Daegu Gyeongbuk Institute of Science and Technology (DGIST)Daegu, South Korea

**Keywords:** ER Ca^2+^, ryanodine receptors, IP**_3_** receptors, autophagy, programmed cell death, autophagic cell death, neural stem cell, insulin withdrawal

## Abstract

Cytoplasmic Ca^2+^ actively engages in diverse intracellular processes from protein synthesis, folding and trafficking to cell survival and death. Dysregulation of intracellular Ca^2+^ levels is observed in various neuropathological states including Alzheimer’s and Parkinson’s diseases. Ryanodine receptors (RyRs) and inositol 1,4,5-triphosphate receptors (IP_3_Rs), the main Ca^2+^ release channels located in endoplasmic reticulum (ER) membranes, are known to direct various cellular events such as autophagy and apoptosis. Here we investigated the intracellular Ca^2+^-mediated regulation of survival and death of adult hippocampal neural stem (HCN) cells utilizing an insulin withdrawal model of autophagic cell death (ACD). Despite comparable expression levels of RyR and IP_3_R transcripts in HCN cells at normal state, the expression levels of RyRs—especially RyR3—were markedly upregulated upon insulin withdrawal. While treatment with the RyR agonist caffeine significantly promoted the autophagic death of insulin-deficient HCN cells, treatment with its inhibitor dantrolene prevented the induction of autophagy following insulin withdrawal. Furthermore, CRISPR/Cas9-mediated knockout of the RyR3 gene abolished ACD of HCN cells. This study delineates a distinct, RyR3-mediated ER Ca^2+^ regulation of autophagy and programmed cell death in neural stem cells. Our findings provide novel insights into the critical, yet understudied mechanisms underlying the regulatory function of ER Ca^2+^ in neural stem cell biology.

## Introduction

A subtle balance between cell survival and death is maintained through intricate networks of molecular signaling machinery. Ca^2+^ is a critical intracellular signal that regulates many cellular processes in development stages of embryo, neuronal proliferation and cognitive ability in the brain (Webb and Miller, 2003; Rosenberg and Spitzer, 2011; Bading, 2013). Due to its versatile nature, molecular mechanisms of Ca^2+^-dependent signaling exist in all types of mammalian cells, and its malfunction fails networks of intracellular signals that maintain cellular homeostasis and cell survival. For instance, Ca^2+^ possesses dual roles in determination of cell fate including both the sustenance of cell viability (Yano et al., 1998) and the activation of cell death machinery (Alberdi et al., 2010), depending on cellular contexts. In the field of programmed cell death (PCD), Ca^2+^ regulation of PCD has been widely investigated in various pathological contexts. Accumulating evidence suggests that an intimate relationship between the perturbed intracellular Ca^2+^ distribution and PCD underlies the pathophysiology of various neural diseases, such as Alzheimer’s (Guo et al., 1996) and Parkinson’s (Gandhi et al., 2009). The importance of Ca^2+^ regulation in neuronal cell death has led to the Ca^2+^ hypothesis in the pathogenesis of Alzheimer’s disease (AD), first proposed by Khachaturian in 1980s (Khachaturian, 1987, 1994). The Ca^2+^ hypothesis states that the persistent upregulation of Ca^2+^ signaling in the hippocampus is responsible for neuronal death and progressive decline in cognition and memory (Guo et al., 1996; Palop et al., 2003; Du et al., 2008) commonly observed in AD.

Apoptosis, necrosis, and autophagic cell death (ACD) are three major modes of PCD which are classified by distinct morphological and biochemical features along with key molecular regulators (Edinger and Thompson, 2004). Despite the unique characteristics among the modes of PCD, Ca^2+^ is important in all three, mostly functioning as an upstream trigger of cell death pathways (Zhivotovsky and Orrenius, 2011). In particular, ryanodine receptors (RyRs) and inositol 1,4,5-triphosphate receptors (IP_3_Rs), the main Ca^2+^ release channels in endoplasmic reticulum (ER), have been implicated in Ca^2+^-mediated regulation of PCD (Mori et al., 2005; Kasri et al., 2006). Compared to apoptosis and necrosis, the exact regulatory function of intracellular Ca^2+^ in ACD has not yet been comprehensively elucidated.

Regulation of autophagy by intracellular Ca^2+^ has been shown to occur in several cell lines and pathological models. Autophagy, meaning “*self-eating*” in Greek, is a cellular process responsible for degradation of cytosolic proteins and subcellular organelles in the lysosomes (Klionsky, 2004). Target constituents and organelles are sequestered in double-membrane vesicles termed autophagosomes and degraded by lysosomal enzymes upon fusion of autophagosomes and lysosomes. Autophagy is generally known as a cytoprotective process which converts long-lived proteins or damaged organelles into metabolic intermediates to counter cellular stress and maintain cellular homeostasis (Dunn, 1994). However, as in the case of ACD, autophagy can function as the primary mode of cell death (Yu et al., 2008; Clarke and Puyal, 2012). ACD is defined as a non-apoptotic PCD *by* or *through* autophagy as its name suggests (Shen and Codogno, 2011). Interestingly, debate remains as to the exact function of intracellular Ca^2+^ in control of autophagy; two opposite views exist based on conflicting reports suggesting both stimulatory and inhibitory roles for Ca^2+^ in autophagy (Criollo et al., 2007; Hoyer-Hansen et al., 2007; Gao et al., 2008; Harr et al., 2010).

We have previously established the cellular model of ACD in primary cultured adult hippocampal neural stem/progenitor (HCN) cells following insulin withdrawal (Yu et al., 2008). Several molecular mechanisms underlying interactions between apoptosis and autophagy, and regulation of PCD in neural stem cells (NSCs) were identified utilizing the insulin withdrawal model of ACD (Yu et al., 2008; Baek et al., 2009; Chung et al., 2015; Ha et al., 2015). NSCs, by definition, feature the multipotency to proliferate and differentiate into different types of neural lineage in the nervous system, and the self-renewal capability to maintain the stem cell population (Gage, 2000). As such, HCN cells have intact differentiation competence as* bona fide* neural stem/progenitor cells (data not shown) with the homogenous expression of neural stem/progenitor marker, nestin (Yu et al., 2008). PCD functions as a rigid quality control mechanism to eliminate faulty or superfluous cells and thereby maintain the integrity and size of the NSC population (Lindsten et al., 2003). The unique properties of NSCs ensure generation of normal tissues in the brain during development and even in adult stages (Oppenheim, 1991; Biebl et al., 2000). Conversely, abnormal functions in NSC physiology may render them largely susceptible to pernicious consequences. For instance, dysregulation in cell cycle, neuronal differentiation, or cell death of NSCs may result in neuronal loss through neurodegeneration and may eventually deteriorate higher cognitive functions (Yamasaki et al., 2007). Therefore, understanding the mechanisms governing survival and death of NSCs is pivotal for the development of therapeutic designs utilizing endogenous NSCs, especially in regard to counter aging and neurodegenerative diseases.

Insulin withdrawal drove the mode of cell death towards ACD in HCN cells despite their intact apoptotic capabilities (Yu et al., 2008; Ha et al., 2015). Of particular interest, we observed a rise in intracellular Ca^2+^ level in insulin-deprived HCN cells (denoted as I(−) HCN cells with their counterpart grown in insulin-containing normal condition as I(+) HCN cells, hereafter; Chung et al., 2015). Since high intracellular Ca^2+^ can promote or suppress autophagy induction depending on cell types and stress context (East and Campanella, 2013), we wondered whether intracellular Ca^2+^ levels impact on the default ACD in I(−) HCN cells. To test this idea, we targeted RyRs and IP_3_Rs, two well-known ER Ca^2+^ channels as the potential route of intracellular Ca^2+^ rise. Here, we observed that a rise in intracellular Ca^2+^ levels occurred mainly through type 3 RyRs (RyR3) rather than IP_3_Rs, and this rise augmented ACD in HCN cells. Our findings can provide a novel insight into the Ca^2+^-mediated regulation of PCD in NSCs and the potential role of RyR3 as a novel molecular target for treatment of neurodegenerative diseases by stem cell therapies.

## Materials and Methods

### Cell Culture

All procedures for the care and use of laboratory animals were approved by the Institutional Animal Care and Use Committee (IACUC) at Daegu Gyeongbuk Institute of Science and Technology (DGIST). Adult rat HCN cells were isolated from the hippocampus of 2-month old Sprague Dawley rats and cultured as previously reported (Chung et al., 2015). Cells were maintained in chemically defined serum-free medium containing Dulbecco’s modified Eagle’s Medium/F-12 supplemented with N2 components and basic fibroblast growth factor (20 ng/ml). Insulin was omitted to prepare insulin-deficient medium. Insulin-containing and insulin-deficient media are denoted as I(+) and I(−), respectively, in this study.

### Pharmacological Reagents

The pharmacological reagents used were prepared at the indicated stock concentrations as follows: Caffeine (C0750; Sigma-Aldrich, St. Louis, MO, USA) was prepared in I(−) medium at 75 mM. IP_3_ (60960; Cayman Chemical, Ann Arbor, MI, USA) was diluted in phosphate-buffered saline (PBS) at 5 mM and dantrolene (14663-23-1; Sigma-Aldrich) was liquefied in dimethyl sulfoxide at 20 mM.

### Cell Death Assay

HCN cells were seeded in a 96-well plate at a density of 5 × 10^4^ cells per cm^2^. Cell death was assayed using Hoechst 33342 (H3570; Invitrogen, Carlsbad, CA, USA) and propidium iodide (P4170; Sigma-Aldrich) staining. In the treatment, each reagent did not exceed 0.5% of the total volume of medium to prevent potential cytotoxicity. Cells were imaged under a fluorescence microscope (Axiovert 40 CFL; Carl Zeiss, Jena, Germany) and collected images were further analyzed for cell counting using ImageJ Software (NIH, Bethesda, MD, USA). Cell death rate was calculated as follows:

Cell death (%) = [PI-positive (dead) cell number/Hoechst-positive (total) cell number] × 100.

### Western Blot Analysis

Methods used for Western blot analysis were similar to those described previously (Yu et al., 2008). HCN cells were harvested in cold PBS at the indicated time points and pellets were then lysed on ice in radioimmunoprecipitation assay buffer (Sigma-Aldrich) containing 1× protease and phosphatase cocktail inhibitors (87786; Thermo Scientific, Waltham, MA, USA) for 30 min. Following centrifugation at 12,000 g for 15 min, protein concentrations were determined using BCA protein assay kit (23224; Thermo Scientific) and protein samples were boiled for 3–5 min at 100°C and separated by SDS-PAGE. The proteins were electrotransferred onto polyvinylidene fluoride membrane with a semi-dry electrophoretic transfer cell (Bio-Rad, Hercules, CA, USA) and the membranes were then blocked for 1 h at room temperature in a blocking buffer consisting of 5% nonfat dry milk and 0.1% Tween 20 in Tris-buffered saline. The membranes were then incubated overnight at 4°C with one of the following primary antibodies: microtubule-associated protein 1A/1B-light chain 3B (LC3B; L7543; Sigma-Aldrich), SQSTM1/p62 (#5114S; Cell Signaling Technology, Danvers, MA, USA) or β-actin (#4967S; Cell Signaling Technology). The primary antibodies were diluted according to the manufacturers’ recommendations. The membranes were washed three times for 10 min each, followed by 1 h incubation at room temperature with peroxidase-conjugated secondary antibodies diluted in blocking solution. After washing, the membranes were then processed for analysis using a chemiluminescence detection kit (34080; Thermo Scientific).

### Real-Time Quantitative PCR

Total RNA was isolated from cells using the ImProm-II Reverse Transcriptase kit (A3803; Promega, Madison, WI, USA) and reverse-transcribed into cDNA. Real-time quantitative PCR was performed with the CFX96 Real-Time System (Bio-Rad) and iTaq Universal SYBR Green Supermix (Bio-Rad). Results were analyzed using MS Excel 2013 (Microsoft, Seattle, WA, USA). β-Actin was used as the reference gene for normalization since its mRNA level did not differ between I(+) and I(−) conditions. Primers used for isoforms of RyRs and IP_3_Rs are listed in Table 1.

**Table 1:** **A list of primers used for quantitative real-time PCR analysis**.

Target	Gene	Forward primer (5′-3′)	Reverse primer (5′-3′)
β-Actin	ACTB	AGCCATGTACGTAGCC	CTCTCAGCTGTGGTGGTGAA
		ATCC
RyR1	RYR1	CTTAACCAGCTCAGACAC	GATTCGACCCAGGTATGGTTC
		CTTC
RyR2	RYR2	GGACTTGACATCCTCTG	GGTTGGAAGTAGTTGAGG
		ACAC	ACAC
RyR3	RYR3	TGGGACAAGTTCGTGAAG	GCCCATGGCAATATCCAA
IP_3_R1	ITPR1	TGTATGCAGAGGGATCTA	TCATCCAACGTCACTCTC
IP_3_R2	ITPR2	AGGGCTCGGTCAATGGCT	CTTCGTTCCCTGCAGCATCC
IP_3_R3	ITPR3	CCAGCTTTCTTCACATCG	CAGCCGCTTGTTCACCGTTAAG

*A list of forward and reverse primers for rat RyR and IP_3_ R isoforms*.

### Immunofluorescence-Based Ca^2+^ Imaging

**Figure 1:**
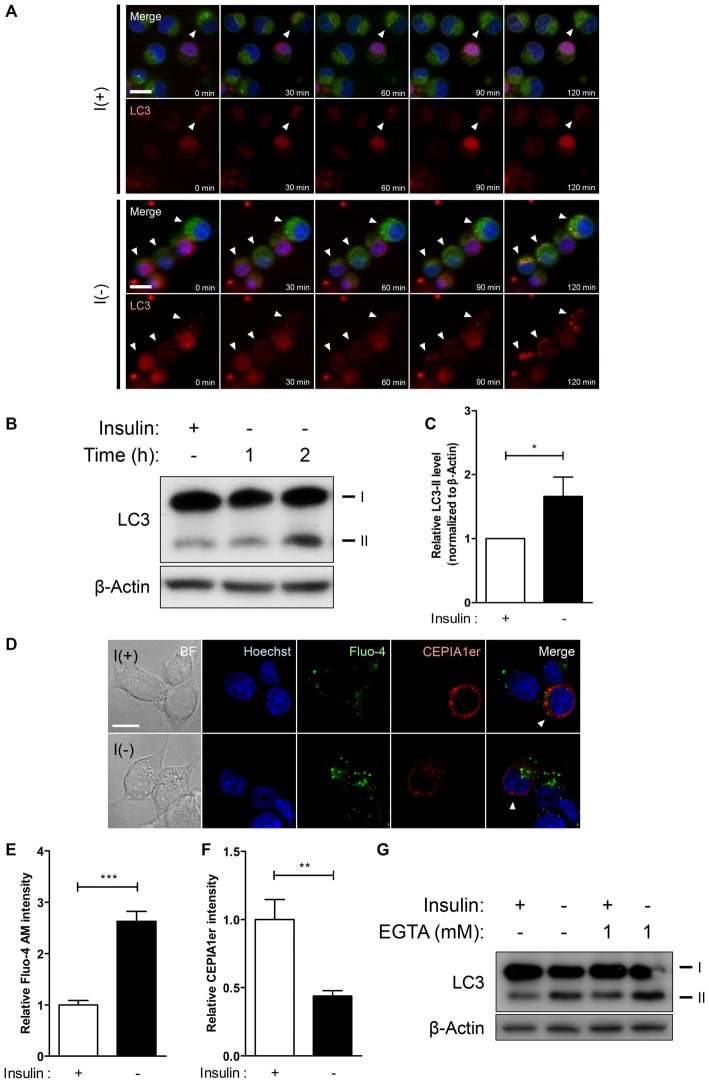
**Insulin withdrawal increases intracellular Ca^2+^ levels without the involvement of extracellular Ca^2+^ in hippocampal neural stem (HCN) cells. (A–C)** Time-course of autophagic induction upon insulin withdrawal was assessed in HCN cells. **(A)** Live-imaging analysis of RFP-LC3-expressing HCN cells stained with Lysotracker Green. Cells indicated by arrowheads show LC3 puncta co-localized with lysosomes. Scale bar, 10 μm.** (B)** Western blot analysis of HCN cells following insulin withdrawal for 1 h and 2 h. The signal intensity of LC3-II was quantified in **(C)** using the ImageJ software and normalized to β-actin (*n* = 4). **(D–F)** Levels of intracellular and endoplasmic reticulum (ER) Ca^2+^ in HCN cells were assessed using Fluo-4 AM and pCMV R-Ca^2+^-measuring organelle entrapped protein indicator 1 in the ER (CEPIA1er), respectively. **(D)** Immunofluorescence analysis of Fluo-4 AM and pCMV R-CEPIA1er in HCN cells under I(+) and I(−) conditions for 24 h. Cells subjected to analysis are indicated by arrowheads. Scale bar, 10 μm. Fluorescence intensities of Fluo-4 AM **(E)** and CEPIA1er **(F)** in HCN cells (*n* = 30 for I(+), 36 for I(−)). **(G)** Western blot analysis of a biochemical marker of autophagy LC3 in HCN cells cultured in I(+) and I(−) conditions with or without EGTA (1 mM) for 6 h. The bars represent the mean ± standard error of the mean (SEM); *p < 0.05, **p < 0.01, ***p < 0.001.

HCN cells were first cultured overnight in 6-well plates at a density of 1 × 10^5^ cells/well in medium without penicillin/streptomycin. On the following day, Lipofectamine 2000 (11668027; Invitrogen)-mediated transfection of pCMV R-CEPIA1er (58216; Addgene, Cambridge, MA, USA) was performed according to the manufacturer’s instruction. Cells were replated on 18 mm round glass coverslips placed in a 12-well plate at 24 h post-transfection and insulin withdrawal or treatment of reagents were applied. At 48 h post-transfection, imaging procedures were proceeded accordingly following each experimental design. For labeling intracellular Ca^2+^, cells were incubated with Fluo-4 AM reagent (F14201; Molecular Probes, Eugene, OR, USA) at 3 μM for 40 min at 37°C. ER Ca^2+^ was assayed by detection of Ca^2+^-measuring organelle-entrapped protein indicator 1 in the ER (CEPIA1er). To monitor intracellular Ca^2+^ and ER Ca^2+^ simultaneously, as depicted in Figures 1D, 3H, 5A, HCN cells transfected with pCMV R-CEPIA1er construct were stained with Fluo-4 AM at 48 h post-transfection. For selective analysis of ER Ca^2+^ expressions in autophagy-induced HCN cells, co-transfection of pCMV R-CEPIA1er and pEGFP-LC3 (21073; Addgene) constructs were performed as shown in Figure 6A. Images were obtained with a confocal microscope (LSM700; Carl Zeiss).

ImageJ (NIH) was used to determine the fluorescence intensities of Fluo-4 AM and CEPIA1er signals. Representative images collected under the same microscope settings were compiled without any modification, and adjusted for brightness and contrast using ImageJ. Background of each summed image was subjected to threshold filtering, and thereby the pixels brighter than a given threshold background value were considered as the signals attributable to the fluorescent Ca^2+^ indicators.

### Live Cell Imaging

Time-lapse live cell imaging analysis of autophagy induction was performed on a Zeiss LSM7 Live laser-scanning confocal microscope (Carl Zeiss). HCN cells were transfected with RFP-LC3 construct (21075; Addgene) and stained with LysoTracker Green DND-26 (L7526; Molecular Probes). The images were acquired every 30 min for 2 h with ZEN Software (Carl Zeiss). Delivery of RFP-LC3 by Lipofectamine 2000 and analysis of obtained images were performed as described above.

### Autophagic Flux Assay

HCN cells expressing mRFP-GFP-LC3 tandem construct were plated on glass coverslips in 12-well plates at a density of 2.0 × 10^5^ cells/ml. The HCN cells were fixed in 4% paraformaldehyde solution for 5 min at room temperature. After washing with PBS twice, the cells were mounted on slides with Mount solution (S3023; Dako, Glostrup, Denmark) and images were obtained with a confocal microscope (LSM700; Carl Zeiss). For delivery of mRFP-GFP-LC3 tandem construct, we followed the transfection procedure described above. The mRFP-GFP-LC3 construct was a kind gift from Dr. Eun-Kyoung Kim (DGIST).

### Generation of CRISPR/Cas9-Mediated RYR3 Knockout HCN Cells

Guide RNAs for gene inactivation of RyR3 were designed and purchased from ToolGen (Seoul, Republic of Korea). HCN cells were transfected with Cas9- and gRNA-encoding plasmids using a Lipofectamine 2000 transfection reagent (Invitrogen) according to the manufacturer’s protocol. Homogeneity of RYR3 knockout was achieved by hygromycin selection (Cat. # ant-hg-1; InvivoGen, San Diego, CA, USA) at 24 h post-transfection, followed by a complete medium change.

### Statistical Analysis

Data are presented as mean ± standard error of the mean (SEM), quantified from at least three independent experiments. Statistical significance was determined using the paired *t*-test for two-group experiments. For experiments with three or more groups, data were compared and analyzed by using one-way analysis of variance (ANOVA) and Tukey’s test. Differences were considered statistically significant when *p* < 0.05.

## Results

### ER-to-Cytosol Ca^2+^ Efflux is Increased Following Insulin Withdrawal in HCN Cells

Our prior studies on autophagic death of insulin-deprived adult rat HCN cells have identified multiple upstream regulators of autophagy and PCD (Yu et al., 2008; Baek et al., 2009; Chung et al., 2015; Ha et al., 2015). The discovery of calpain as a molecular determinant of PCD modes has hinted that intracellular Ca^2+^ signaling may underlie the interconnection of cell death pathways upon insulin removal in HCN cells (Chung et al., 2015). Through live-imaging and Western blot analyses we found that absence of insulin gradually induces autophagic response in HCN cells as early as 2 h after insulin withdrawal, since live cell imaging with Lysotracker Green and RFP-LC3 at 30’, 60’, 90’, and 120’ showed gradual appearance of LC3 puncta co-localized with the lysosomes (Figure 1A). An increased conversion of LC3-I to LC3-II was also evident from 1 h (Figures 1B,C). To monitor changes in Ca^2+^ dynamics, we measured the intracellular and ER-specific Ca^2+^ levels in I(−) HCN cells using the intracellular Ca^2+^ indicator Fluo-4 AM and a genetically encoded ER Ca^2+^ indicator CEPIA1er, respectively (Figure 1D). While the intracellular Ca^2+^ level in insulin-deprived HCN cells was two-fold greater than control cells, the fluorescence intensity of CEPIA1er revealed that ER Ca^2+^ levels were halved following insulin withdrawal (Figures 1E,F). We next tested whether autophagy regulation in HCN cells is mainly achieved by intracellular Ca^2+^ dynamics or involves extracellular Ca^2+^ influx. A specific Ca^2+^ chelator EGTA was added to restrict availability of extracellular Ca^2+^ from HCN cells. We found that presence of EGTA for 6 h affected neither autophagy induction (Figure 1G) nor the subsequent cell death in both I(+) and I(−) conditions (data not shown), indicating that autophagy in HCN cells is modulated mainly through intracellular Ca^2+^ events. Autophagy induction was assessed based on the increased modification of LC3-I into LC3-II. Conversion of LC3-I (18 kDa) to LC3-II (16 kDa) occurs after lipidation in autophagosomes, therefore an increase of LC3-II indicates the increase in the number of autophagosomes (Ichimura et al., 2000). Accordingly, LC3 has been well established as a marker of autophagic flux in I(−) HCN cells in our previous reports (Yu et al., 2008; Baek et al., 2009; Chung et al., 2015; Ha et al., 2015).

### RyR3 is the Major RyR Isoform Expressed in HCN Cells and its Expression is Elevated Following Insulin Withdrawal

**Figure 2:**
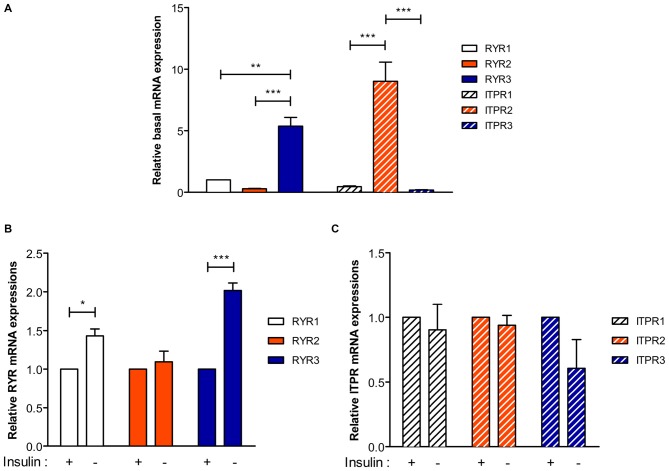
**Type 3 Ryanodine receptor (RyR) is the major isoform in HCN cells and its expression is upregulated following insulin withdrawal. (A)** Real-time quantitative PCR analysis of basal expression levels of RyR and IP_3_R genes denoted as RYR and ITPR, respectively, after normalization to β-actin. **(B,C)** A change in the mRNA levels of RYR **(B)** and ITPR **(C)** 24 h following insulin withdrawal. Each set of experiment was conducted in triplicate per experiment (*n* = 4). The bars represent the mean ± SEM; *p < 0.05, **p < 0.01, ***p < 0.001.

Since insulin withdrawal-induced autophagy in HCN cells accompanies altered levels of ER Ca^2+^, we first characterized expression patterns of RyRs and IP_3_Rs, the main Ca^2+^ release channels in ER membranes. Real-time quantitative PCR analysis revealed that genes encoding type 3 RyR (RYR3) and type 2 IP_3_R (ITPR2) were the most abundantly expressed RyR and IP_3_R transcripts in HCN cells at the basal state, respectively (Figure 2A). Interestingly, insulin withdrawal for 24 h enhanced the expression of RYR1 and RYR3 with more significant increase of RYR3 while genes encoding IP_3_Rs were unaffected (Figures 2B,C). Given the abundance of RYR3 and its substantial upregulation following insulin withdrawal, these data suggest a potential role of RyR3 in the modulation of autophagy in HCN cells.

### A RyR Agonist Caffeine Further Promotes ACD in Insulin-Deprived HCN Cells

**Figure 3:**
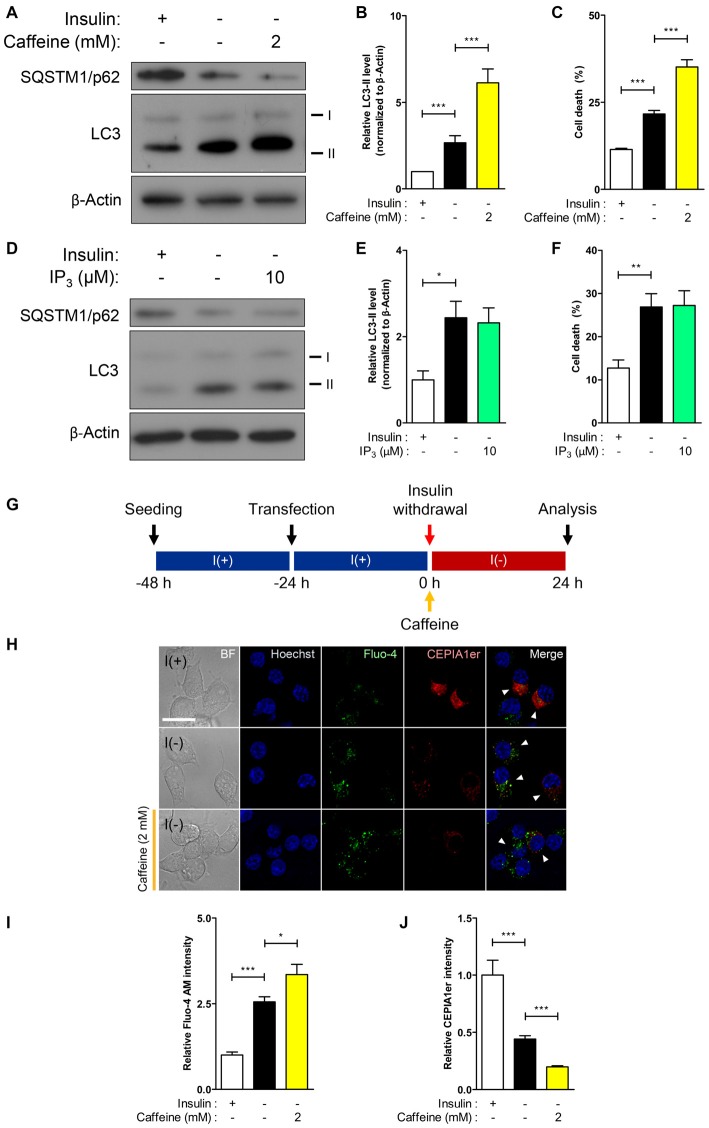
**RyR agonist caffeine potentiates autophagic cell death (ACD) in I(−) HCN cells. (A–C)** Effect of caffeine on autophagy and cell death was analyzed in HCN cells. **(A)** Western blot analysis of the autophagy marker proteins LC3 and SQSTM1/p62 in I(−) HCN cells treated with caffeine for 24 h. The signal intensity of LC3-II was quantified in **(B)** using the ImageJ software and normalized to β-actin (*n* = 7). **(C)** Rate of cell death measured in parallel sets of experiments as conducted in **(B)**. **(D–F)** Analysis of inositol 1,4,5 triphosphate (IP_3_) effect on autophagy and cell death in I(−) HCN cells treated with IP_3_ for 24 h. **(D)** Western blot analysis of LC3 and SQSTM1/p62 in I(−) HCN cells treated with IP_3_. The signal intensity of LC3-II was quantified in **(E)** using the ImageJ software and normalized to β-actin (*n* = 7). **(F)** Rate of cell death measured in parallel sets of experiments conducted in **(E)**. **(G–J)** Levels of intracellular and ER Ca^2+^ in I(−) HCN cells treated with caffeine for 24 h were measured by Fluo-4 AM and pCMV R-CEPIA1er, respectively. **(G)** An experimental scheme for immunofluorescence-based analysis of Ca^2+^ in HCN cells. **(H)** Immunofluorescence images of Fluo-4 AM and pCMV R-CEPIA1er in caffeine-treated I(−) HCN cells compared to I(−) alone or I(+) cells. Cells subjected to analysis are indicated by arrowheads. Scale bar, 10 μm. **(I,J)** Fluorescence intensities of Fluo-4 AM **(I)** and CEPIA1er **(J)** were quantified (*n* = 40 for I(+), 36 for I(−), 47 for I(−)/Caffeine). The bars represent the mean ± SEM; *p < 0.05, **p < 0.01, ***p < 0.001.

Dramatic elevation of RyR transcripts observed in I(−) HCN cells compared to control I(+) cells prompted us to investigate the functional role of RyRs with respect to autophagy activation. Stemming from our results in Figure 2, we hypothesized that RyRs are more profoundly associated with insulin withdrawal-induced ACD in HCN cells than IP_3_Rs. As expected, treatment with the RyR agonist caffeine enhanced autophagic activity shown by the heightened level of the autophagy marker LC3-II in I(−) HCN cells experiencing ACD (Figures 3A,B). A reduction in the levels of SQSTM1/p62, an adaptor protein which recognizes and loads cargo proteins or organelles subjected for autophagic degradation into autophagosomes, also reflects the promotion of autophagy by caffeine (Figure 3A). The increased rate of cell death in caffeine-treated cells demonstrates that the furtherance of autophagy-dependent cell death mechanism remains intact in HCN cells (Figure 3C). However, an IP_3_R agonist IP_3_ did not increase autophagic activity (Figures 3D,E). Likewise, treatment with IP_3_ failed to increase the rate of cell death in I(−) HCN cells (Figure 3F). To examine whether the caffeine-induced potentiation of ACD involves ER-to-cytosol Ca^2+^ transfer, we measured the signal intensities of Fluo-4 AM and CEPIA1er following the presented experimental scheme (Figures 3G,H). As expected, addition of caffeine in I(−) HCN cells led to greater elevation and reduction in fluorescence intensities of Fluo-4 AM and CEPIA1er compared with I(−) alone, respectively (Figures 3I,J). Thus, addition of caffeine further enhanced ACD in I(−) HCN cells by accelerating the release of Ca^2+^ from the ER.

### ACD Induction by Caffeine is Precluded in Autophagy-Defective HCN Cells Depleted of Atg7

**Figure 4:**
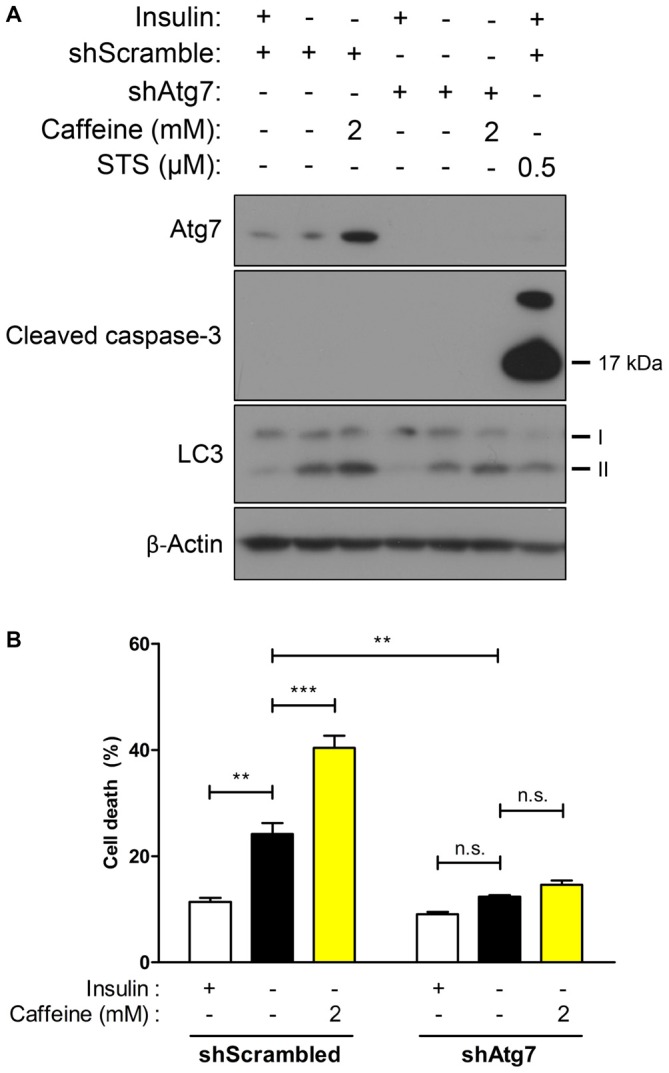
**Caffeine-induced potentiation of ACD is prevented in Atg7 knockdown HCN cells. (A,B)** Effect of caffeine on autophagic activity was assessed in autophagy-defective HCN cells generated by shRNA-mediated suppression of Atg7 gene. **(A)** Western blot analysis of autophagy- and apoptosis-related proteins after caffeine treatment for 24 h in I(−) HCN cells depleted of Atg7. Staurosporine (STS) was treated for 10 h before cell harvest to induce caspase-3 activation as a positive control of apoptosis.** (B)** Cell death analysis in caffeine-treated Atg7 knockdown HCN cells at 24 h. Each experiment set was performed in triplicate per experiment (*n* = 4). The bars represent the mean ± SEM; **p < 0.01, ***p < 0.001; n.s., non-significant.

Due to accelerated autophagic activity and the subsequent cell death induced by caffeine in I(−) HCN cells, we characterized its effect in the absence of autophagy to confirm the requirement of autophagy for the action of caffeine. Autophagy-related genes (Atg) initiate autophagosome formation through Atg12-Atg5 and LC3-II complexes (Mizushima et al., 1998). Atg7 is required for Atg12-Atg5 conjugation and LC3 lipidation (Ohsumi, 2001) and we generated Atg7 stable knockdown HCN cells by genetically suppressing Atg7 expression with the lentivirus expressing Atg7-targeting shRNA. As shown in Figure 4A, following insulin withdrawal, autophagy—depicted by LC3-II levels—was reduced in shAtg7-transduced HCN cells compared to cells transduced with control shScramble. Interestingly, 2 mM caffeine treatment did not significantly elevate cell death in autophagy-defective HCN cells while the rate of cell death in I(−) scramble controls was robustly increased (Figure 4B). Caffeine treatment in I(−) cells did not involve activation of caspase-dependent apoptosis, indicating that caffeine-induced cell death is a continuance of a genuine ACD in I(−) HCN cells (Figure 4A).

### Autophagy is Diminished by Pharmacological or Genetic RyR Inhibition in I(−) HCN Cells

**Figure 5:**
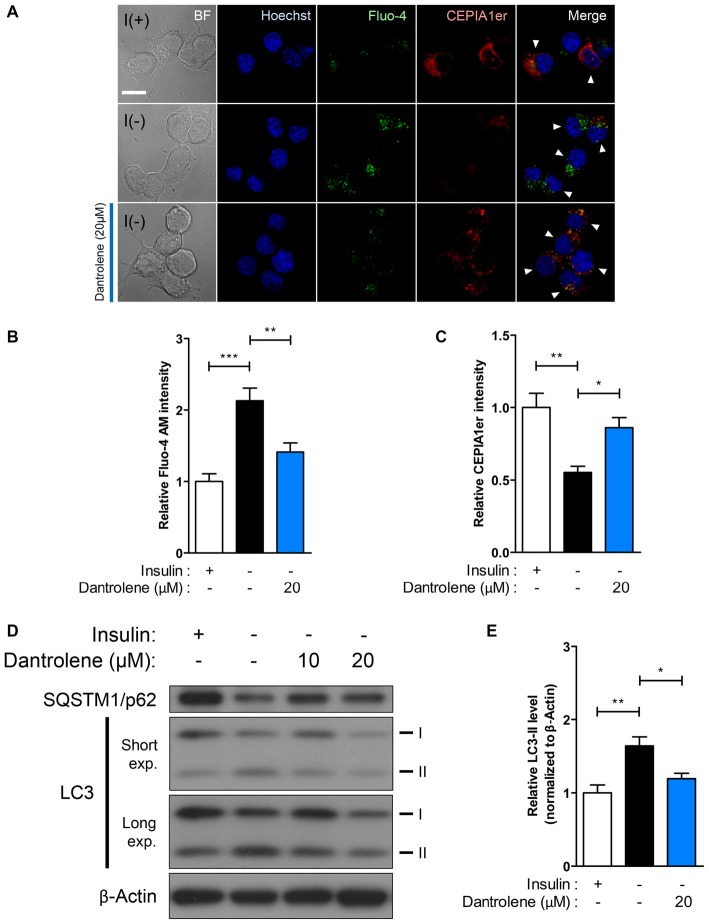
**RyR inhibition suppresses ACD in HCN cells. (A–E)** Treatment with the RyR antagonist dantrolene inhibits autophagic activity in HCN cells. **(A)** Immunofluorescence-based detection of Fluo-4 AM and pCMV R-CEPIA1er in I(−) HCN cells treated with dantrolene. Cells subjected to analysis are indicated by arrowheads. Scale bar, 10 μm. Fluorescence intensities of Fluo-4 AM **(B)** and CEPIA1er **(C)** in HCN cells were quantified (*n* = 22 for I(+), 30 for I(−), 27 for I(−)/Dantrolene). **(D)** Western blot analysis of LC3 and SQSTM1/p62 in I(−) HCN cells treated with dantrolene for 6 h. The signal intensity of LC3-II was quantified in **(E)** using the ImageJ software and normalized to β-actin (*n* = 6). The bars represent the mean ± SEM; *p < 0.05, **p < 0.01, ***p < 0.001.

Our results on potentiation of autophagic death of I(−) HCN cells through stimulation of Ca^2+^ release by the RyR agonist caffeine provoked us to test whether inhibition of RyR reverses the autophagic events in I(−) HCN cells. To examine the effect of RyR inhibition on autophagy, we treated I(−) HCN cells with dantrolene, a RyR antagonist (Kobayashi et al., 2005). RyR inhibition by dantrolene effectively prevented Ca^2+^ release from the ER, as shown by the reversal of the changes in fluorescence intensities of Fluo-4 AM and CEPIA1er observed in I(−) HCN cells (Figures 5A–C). Thereby, dantrolene significantly reduced the level of LC3-II proteins in a concentration-dependent manner (Figures 5D,E).

**Figure 6:**
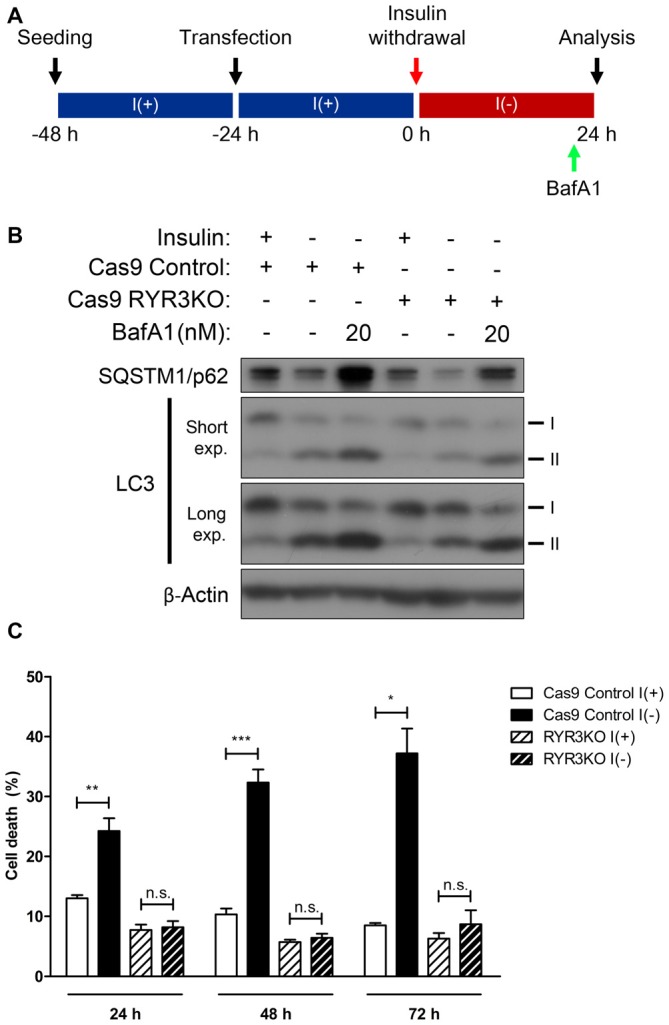
**Genetic suppression of RyR3 diminishes autophagic flux in I(−) HCN cells. (A–C)** Effect of CRIPR/Cas9-induced RYR3 knockout on ACD in I(−) HCN cells. **(A)** An experimental scheme for inhibition of autophagic flux by BafA1 treatment. **(B)** Characterization of autophagic flux by immunoblotting of LC3 and SQSTM1/p62 in RYR3KO I(−) HCN cells treated with BafA1. Cells were treated with BafA1 for 2 h at given concentration before harvest. **(C)** Rate of cell death in RYR3KO HCN cells under I(+) and I(−) conditions for 24 h, 48 h, and 72 h; each experiment performed in triplicate per experiment (*n* = 4). The bars represent the mean ± SEM; *p < 0.05, **p < 0.01, ***p < 0.001; n.s., non-significant.

Of the RyR isoforms in HCN cells, RyR3 is the most prominent, and following insulin withdrawal, the mRNA of this isoform showed the largest increase as well (Figures 2A,B). Along with the altered expression of RyR3 transcripts, the observation that autophagy is modulated by RyR agonist and antagonist suggests that ACD may be regulated via RyR3-mediated intracellular Ca^2+^ signaling in HCN cells. Thus, we generated RYR3 knockout (RYR3KO) HCN cells utilizing CRISPR/Cas9-mediated gene inactivation. Consistent with our hypothesis, the prominence of autophagy—as measured by LC3-II—was substantially decreased in I(−) HCN cells absent of RyR3 (Figure 6B). Furthermore, we used bafilomycin A1 (BafA1), a specific inhibitor of late phase autophagy that acts by preventing formation of autolysosomes, to further verify the attenuated autophagic flux in RYR3KO (Figure 6A). Robust accumulation of LC3-II proteins due to the inhibition of autophagic flux by BafA1 indicates a rapid conversion of LC3-I to LC3-II, reflective of high autophagic flux (Yamamoto et al., 1998). The accumulation of LC3-II in RYR3KO I(−) HCN cells by BafA1 treatment was substantially diminished compared to BafA1-treated I(−) control cells (Figure 6B). Consistent with the reduced autophagic flux in RYR3KO HCN cells, RyR3 depletion significantly reduced the insulin withdrawal-induced cell death in HCN cells up to 72 h (Figure 6C).

### Knockout of RyR3 Gene Occludes ER Ca^2+^ Release and Thereby Prevents ACD in I(−) HCN Cells

**Figure 7:**
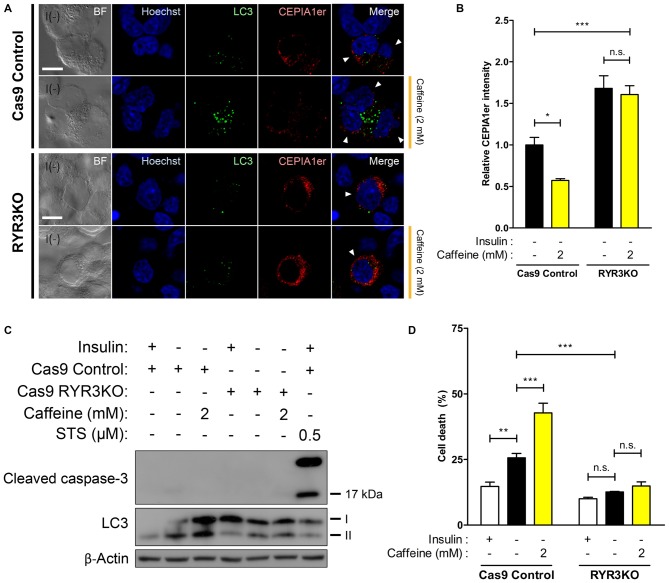
**ER Ca^2+^ efflux is significantly inhibited in I(−) RYR3KO HCN cells. (A,B)** Caffeine was used to provoke ER-to-cytosol transfer of Ca^2+^ in control I(−) and RYR3KO I(−) HCN cells. **(A)** Immunofluorescence images of pCMV R-CEPIA1er in HCN cells experiencing autophagy depicted by puncta formation of EGFP-LC3 24 h following insulin withdrawal. Cells subjected to analysis are indicated by arrowheads. Scale bar, 10 μm. Fluorescence intensity of CEPIA1er **(B)** selectively in EGFP-LC3-positive HCN cells was quantified (*n* = 36 for Cas9 Control I(−), 41 for Cas9 Control I(−)/Caffeine, 32 for RYR3KO I(−), 43 for RYR3KO/Caffeine). The bars represent the mean ± SEM; *p < 0.05, ***p < 0.001; n.s., non-significant.** (C,D)** Caffeine failed to facilitate ACD of I(−) HCN cells in the absence of RyR3. **(C)** Western blot analysis of cleaved caspase-3 and LC3 in I(−) RYR3KO HCN cells treated with caffeine.** (D)** Cell death analysis in RYR3KO HCN cells treated with caffeine for 24 h. Each experiment set was performed in triplicate per experiment (*n* = 6). The bars represent the mean ± SEM; **p < 0.01, ***p < 0.001; n.s., non-significant.

Our results thus far indicate that RyR3 is essential for the regulation of ACD of HCN cells following insulin withdrawal. To further corroborate our hypothesis that a RyR3-mediated rise in intracellular Ca^2+^ level is a primary mechanism inducing autophagy in I(−) HCN cells, we measured Ca^2+^ levels in HCN cells lacking RyR3. Since it was of our specific interest to test RyR3-mediated ER Ca^2+^ regulation of autophagy, we co-assayed for the autophagy marker LC3 instead of Fluo-4 AM along with CEPIA1er for immunofluorescence-based analysis. Puncta formation of fluorescently tagged-LC3 has been commonly used to visualize the cells undergoing autophagy (Chung et al., 2015; Ha et al., 2015). Therefore, co-transfection of pCMV R-CEPIA1er and pEGFP-LC3 constructs allows selective analysis of ER Ca^2+^ levels in autophagy induced-HCN cells at the single cell level (Figure 7A). Quantification of CEPIA1er fluorescence intensity in EGFP-LC3-positive HCN cells revealed no significant ER Ca^2+^ changes provoked by caffeine in RYR3KO HCN cells while caffeine promoted ER Ca^2+^ release in I(−) Cas9 control cells (Figure 7B).

Lastly, we explored whether RYR3KO HCN cells resistant to insulin withdrawal-induced ACD were also impervious to caffeine-mediated induction of autophagy. Western blot analysis revealed that induction of autophagy following caffeine treatment was only visible in Cas9 I(−) control HCN cells, but not in RYR3KO I(−) cells (Figure 7C). Likewise, caffeine potentiated ACD in control I(−) cells, but not in RYR3KO I(−) HCN cells, consistent with our results from Figure 6 (Figure 7D). Since cleaved caspase-3, which reflects the activation of apoptosis, was not detected in either control or RYR3KO HCN cells, it is plausible to conclude that the altered rate of cell death by caffeine was due to ACD, further implicating the prominence of RyR3 in regulation of autophagy in HCN cells.

**Figure 8:**
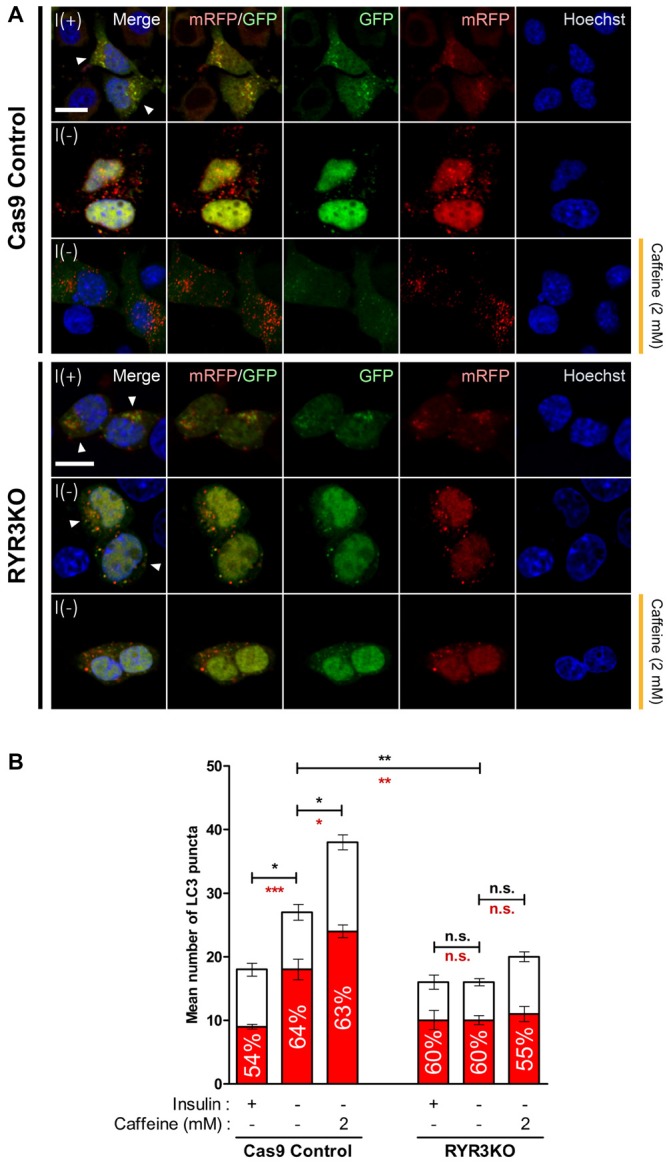
**Autophagic flux in RYR3KO HCN cells is unaltered upon insulin withdrawal or caffeine treatment. (A,B)** Caffeine failed to induce autophagic flux of I(−) HCN cells in the absence of RyR3. **(A)** Immunofluorescence analysis of HCN cells transfected with mRFP-GFP-LC3 tandem construct. Images were analyzed 24 h following insulin withdrawal with or without caffeine treatment. Cells subjected to analysis are indicated by arrowheads. Scale bar, 10 μm. **(B)** Quantification of mRFP- and GFP-LC3 puncta in RYR3KO HCN cells treated with caffeine compared to controls (*n* = 22 for Cas9 Control I(+), 30 for Cas9 Control I(−), 27 for Cas9 Control I(−)/Caffeine, 29 for RYR3KO I(+), 34 for RYR3KO I(−), 25 for RYR3KO/Caffeine). The bars represent the mean ± SEM; *p < 0.05, **p < 0.01, ***p < 0.001; n.s., non-significant. The black and red asterisks indicate the statistical significance in the comparison of total and mRFP puncta numbers, respectively.

To more precisely monitor changes in autophagic flux by caffeine treatment and RyR3 gene inactivation, we utilized monomeric RFP-GFP tandem fluorescent-tagged LC3 (mRFP-GFP tandem LC3). The mRFP-GFP tandem LC3 assay is based on lysosomal quenching of GFP fluorescence but not mRFP fluorescence in cellular compartments with low pH such as inside the lysosome (Bampton et al., 2005). By assessing the percentage of mRFP-only positive LC3 puncta out of total fluorescent-tagged LC3 puncta, we morphologically traced autophagosome maturation and thereby measured autophagic flux in HCN cells (Figure 8A). Cas9 control cells under I(−) condition exhibited significantly greater percentage of mRFP-only positive LC3 puncta compared to I(+) condition; moreover, caffeine further increased the percentage of mRFP-only LC3 punta as well as the total number of LC3 puncta (Figure 8B). These data indicated increased autophagy flux and maturation of autophagic vacuoles into autolysosomes by insulin withdrawal and caffeine treatment. On the contrary, there were no apparent increases in either percentage of mRFP-only LC3 puncta or total LC3 punta numbers in RYR3KO HCN cells, suggesting defective autophagy induction in the absence of RyR3 despite insulin withdrawal or caffeine treatment (Figures 8A,B).

## Discussion

PCD in multipotent NSCs is of immense importance due to its roles in ensuring appropriate brain development and function through the fine regulation of the size and integrity of NSC pools. Many reports have documented the pathophysiology of neurodegeneration by aberrant regulation of the subtle balance between cell survival and death in the brain (Vila and Przedborski, 2003; Krantic et al., 2005). However, the mechanisms underlying PCD of NSCs have been relatively under-investigated compared to a series of extensive studies on neuronal cell death conducted for the past several decades. To this end, albeit investigated at the cellular level, our insulin withdrawal model of ACD in HCN cells has physiological implications especially in relation to AD. Recent studies have delineated that impaired insulin signaling and resistance to insulin or glucose uptake in the brain are strongly associated with the pathogenesis of AD (Talbot et al., 2012; De Felice et al., 2014; Willette et al., 2015). This compelling clinical evidence implicates the autophagy-related cellular processes including ACD due to dysregulated insulin signaling in pathogenic development of AD.

Our previous discovery on calpain as a key negative regulator of ACD and a molecular switch of PCD modes in HCN cells suggests a critical role for intracellular Ca^2+^ in the regulation of programmed death of NSCs (Chung et al., 2015). In this study, we elucidated RyR3-mediated intracellular Ca^2+^ dynamics underlies ACD of HCN cells following insulin withdrawal. A distinct pattern of ER Ca^2+^ change denoted by CEPIA1er, along with the increased intracellular Ca^2+^ levels in I(−) HCN cells, hinted to the possible existence of molecular regulators of intracellular Ca^2+^ dynamics which respond upon insulin withdrawal and subsequently govern autophagy induction. Through real-time quantitative PCR analysis, we assessed the expression levels of RyR and IP_3_R genes, denoted as RYR and ITPR, respectively, the genes encoding for the main ER Ca^2+^ release channels (Figure 2). RYR3 and ITPR2 were identified to be the major isoforms of RyR and IP_3_R, respectively. Intriguingly, only the expression of RyR3 transcripts showed roughly a two-fold increase in response to insulin withdrawal for 24 h while IP_3_R2 gene expression was unaltered, suggesting a more pronounced involvement of RyR3 in insulin withdrawal-induced autophagy in HCN cells. Furthermore, RyR activation by caffeine, but not IP3, elevated ACD in I(−) HCN cells. A series of subsequent experiments utilizing RYR3KO HCN cells further corroborated our notion on the selective modulation of autophagy in HCN cells via RyR3-mediated ER Ca^2+^ efflux.

Lack of acceleration in autophagic flux following caffeine treatment in RYR3 KO I(−) HCN cells were supported by both Western blot and fluorescence-based analyses. Assessment of autophagy flux using a tandem fluorescent-tagged LC3 may warrant caution, because of the occasional aberrant, prolonged stability of GFP in acidic pH (Bampton et al., 2005; Mizushima, 2009). Thus, our imaging analysis was focused on the percentage of mRFP-only positive LC3 puncta out of total fluorescent-tagged LC3 puncta to represent the relative number of lysosome-localized LC3 puncta (Figure 8B).

Our finding on RyR3 regulation of PCD in the absence of insulin signaling is supported by similar results from other studies. Dysregulation of intracellular Ca^2+^ is an underlying component of various neurodegenerative diseases, and recent evidence implicates RyR in the pathology of AD, further highlighting the functional role of RyRs in the aberrant physiological conditions (Supnet et al., 2010; Bruno et al., 2012; Oules et al., 2012; Wu et al., 2013).

Under controlled *in vitro* setting, we were able to examine the regulation of RyR3 in autophagy of HCN cells in the absence of other types of PCD; however, it is unlikely that only a single mode of cell death is activated in the affected brain region under neuropathological situations. Clinical evidence on co-existence of autophagy and apoptosis have been reported in the brains of patients with AD and Parkinson’s diseases (PD; Anglade et al., 1997; Stadelmann et al., 1999). Indeed, crosstalk between autophagy and other PCD modes, mostly apoptosis, has been documented (Eisenberg-Lerner et al., 2009; Chung and Yu, 2013; Chung et al., 2015). Admittedly, the association between RyR3-mediated Ca^2+^ signaling and ACD induction in HCN cells should be perceived as one of several cellular events that could occur *in vivo*. Nevertheless, insulin withdrawal-induced death of HCN cells is regarded as a genuine model of ACD (Shen and Codogno, 2011; Clarke and Puyal, 2012) and can be utilized to study the molecular mechanisms of autophagy and its interrelation with other modes of PCD.

**Figure 9:**
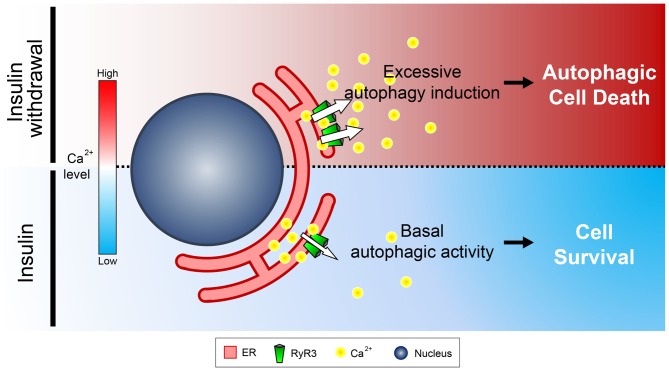
**Schematic diagram illustrating the role of ER-to-cytosol Ca^2+^ in regulation of survival and death of NSCs in insulin withdrawal model.** Presence of insulin constitutively ensures proper distribution of intracellular Ca^2+^ which maintains homeostasis of HCN cells. Inactivation of the pro-survival insulin signaling pathway by insulin withdrawal leads to increased expression and activation of RyR3 which subsequently discharge Ca^2+^ from the ER, the major intracellular Ca^2+^ store. Increase in cytosolic Ca^2+^ subsequently triggers a signaling cascade that induces ACD in HCN cells.

To our knowledge, this is the first report suggesting that RyR3 is the major isoform expressed in NSCs in the hippocampus. Interestingly, mRNA levels of RyR2 were reportedly shown to be substantially greater than other RyR isoforms in mouse hippocampal neurons (Wu et al., 2013). Moreover, a reduced insulin signaling was implicated in neuroprotective autophagy activation in cortical neurons (Young et al., 2009). Connecting these findings, the distinctly pronounced expression pattern of RyR3 may render NSCs to undergo PCD by autophagy instead of neuroprotection. A previous study by Balschun et al. (1999) has delineated impairment of synaptic plasticity in the hippocampi of mice lacking RyR3. Combining our observation of pro-survival effect by RYR3 knockout in NSCs and a seemingly harmful effect in neurons by RyR3 deletion suggest a distinct, cell type-specific role for RyR3. The exact functional implication of differential expression of RyR isoforms in NSCs and neurons remains an enthralling research topic to be investigated. In summary, we unveiled the unique regulatory function of RyR3-mediated intracellular Ca^2+^ on ACD in HCN cells (Figure 9). Our results may provide a profound insight into understanding the intricate mechanisms underlying survival and death of NSCs during developmental stages, neurogenesis, and even pathophysiological contexts.

## Author Contributions

S-WY: conception and design, data analysis and interpretation, manuscript writing, final approval of manuscript. KMC: conception and design, collection and assembly of data, data analysis and interpretation, manuscript writing. E-JJ, HP, H-KA: collection of data.

## Conflict of Interest Statement

The authors declare that the research was conducted in the absence of any commercial or financial relationships that could be construed as a potential conflict of interest.
